# Increasing rates of cervical cancer in young women in England: an analysis of national data 1982–2006

**DOI:** 10.1038/bjc.2011.196

**Published:** 2011-06-07

**Authors:** G Foley, R Alston, M Geraci, L Brabin, H Kitchener, J Birch

**Affiliations:** 1 Cancer Research UK Paediatric and Familial Cancer Research Group, School of Cancer and Enabling Sciences, Stopford Building, University of Manchester, Manchester Academic Health Sciences Centre, Manchester M13 9PT, UK; 2 Academic Unit of Obstetrics and Gynaecology, University of Manchester, School of Cancer and Enabling Sciences, St Mary's Hospital, Manchester Academic Health Sciences Centre, Manchester M13 9WL, UK

**Keywords:** cervical cancer, incidence trends, birth cohorts, cervical screening, young women, HPV vaccination

## Abstract

**Background:**

In England, cervical cancer is the second most common cancer in women aged under 35 years. Overall incidence of cervical cancer has decreased since the introduction of the national screening programme in 1988 but recent trends of incidence in young women have not been studied in detail.

**Methods:**

Information on 71 511 incident cases of cervical cancer in England, 1982–2006, in 20–79-year-olds was extracted from a national cancer registration database. Changes in incidence were analysed by age group, time period and birth cohort. Poisson regression was used to estimate annual percentage change (APC).

**Results:**

Overall incidence, during 1982–2006, fell significantly from 213 to 112 per million person years. However, in 20–29-year-olds, after an initial fall, incidence increased significantly during 1992–2006, (APC 2.16). In 30–39-year-olds incidence stabilised during the latter part of the study period. The pattern was most marked in the North East, Yorkshire and the Humber and East Midlands regions. Birth cohorts that were initially called for screening between 60–64 and 35–39 years of age show an incidence peak soon after the age of presumed first screen, whereas younger birth cohorts show a peak at about 35 years of age. Incidence in the 1977–1981 birth cohort has increased relative to that among women born between 1962 and 1976.

**Conclusion:**

These results have implications for cervical screening, human papilloma virus vaccination and other public health interventions targeting young people.

The incidence of cervical cancer in England in 2007 was 8.0 per 100 000 females, making it the second most common cancer in women under 35 (Office for National Statistics, 2010). Cervical cancer is caused by human papilloma virus (HPV). There are at least 15 high-risk oncogenic strains of HPV but over 70% of cases worldwide are associated with strains 16 and/or 18 (Walboomers *et al*, 1999; Munoz *et al*, 2003). The overall prevalence of HPV infection among women with normal cervical cytology in the UK is 8.9 and 2.4% for HPV 16 and/or 18 (WHO/ICO, 2010). This infection leads to cervical cancer in a small proportion of infected women only. Cervical cancer screening has been available in England since 1967. A formalised national screening programme was established in 1988 for women aged 20–65 years, but in 2005, age of first screening invite was raised to 25 years. Screening uptake and cervical cancer incidence rates have been shown to vary with age, level of education, affluence and ethnicity (Fouquet and Gage, 1996; Baker and Middleton, 2003; Sutton and Rutherford, 2005; Moser *et al*, 2009; NHS Information Centre, 2009). Overall, the incidence of carcinoma of the cervix has decreased in England since 1990 secondary to the national screening programme (Quinn *et al*, 2001).

Recent trends in incidence of cervical cancer in younger women have not been studied in detail. Changes in public health prevention measures, including cervical screening policy and the introduction of the HPV vaccination programme in 2008 (National Health Service, 2008), make examining trends essential for disease control and health commissioning. The present study explores geographical, socioeconomic and age-related trends in incidence of cervical cancer from 1982 to 2006 in England, with emphasis on those aged 20–39 years. The results will provide baseline data for comparison with future trends following the introduction of HPV vaccination and have other important implications for public health policies.

## Methods

Cases of cancer of the cervix diagnosed during the time period 1982–2006 in England were extracted from a database compiled by the National Cancer Intelligence Centre, Office for National Statistics (ONS), London. Cases registered from 1982 to 1994 were coded using the International Classification of Diseases ninth revision (ICD-9) (World Health Organisation, 1977). The ICD tenth revision (ICD-10) was used for cases registered from 1995 to 2006 (World Health Organisation, 1992). Data supplied comprised age in years at diagnosis, year of diagnosis, deprivation index quintile and Government office Region (GOR).

The deprivation index quintile was calculated as follows. For each Lower Super Output Area (LSOA) in England, the Index of Multiple Deprivation (IMD) (Income domain) based on 2001 census data was calculated (Neighbourhood Renewal Unit, 2004). This measures the proportion of the population in households in receipt of means tested benefits and thus measures the proportion of the population suffering from income deprivation. The index was simplified into quintiles. Each case was allocated to an LSOA, and so to a deprivation quintile, on the basis of the postcode of the place of permanent residence at the time of diagnosis. In order to safeguard anonymity, only the deprivation quintile, not the actual value of the IMD or the LSOA of residence, was supplied.

Annual population estimates by single year of age, sex and GOR were obtained from population estimates unit of the ONS. Population estimates for 2001 by 5-year age group, LSOA, GOR and sex were obtained from national census data, as were the deprivation quintiles of the LSOAs. These populations and patient counts were used to calculate incidence rates per million person years (pmpy).

For the analysis of incidence trends, cases were stratified into 5-year age groups, 5-year time periods and, where appropriate, by GOR. Incidence rates were standardised to the European standard population using the direct method (Quinn *et al*, 2005).

Poisson regression was used to estimate the annual percentage change (APC) and its 95% confidence interval and to examine the statistical significance of the changes over time in the APC. This was done by examining the difference in fit between models with a constant APC over the five periods with a model with a different trend in the early periods and later periods. To ensure continuity in the estimate, the time period 1992–1996 was included both as end and starting point of the two consecutive segments of the piecewise linear trend.

Age–period–birth cohort analyses of incidence rates were based on five birth year intervals from 1902–1906 to 1982–1986, with 5-year age groups from 20–24 to 75–79 and 5-year calendar time periods from 1982–1986 up to 2002–2006 to give matching birth cohorts, age groups and time periods. The relationships between age group, birth cohort and time period were estimated using the method of Clayton and Schifflers (1987a, 1987b), which assumes that the number of cases in each cohort/age group/period combination follows a Poisson distribution. The presence of overdispersion was adjusted for using a quasi-poisson model (McCullagh and Nelder, 1989), where appropriate.

Smoothed incidence rates by single year of age were calculated by fitting a generalised additive model with penalised regression splines to the counts (Wood, 2008).

For patients diagnosed from 1997 to 2006, the incidence rates by deprivation quintile were calculated using the data from the 2001 census. Analysis of the variability in incidence by deprivation was conducted both nationally and by GOR, again using Poisson regression.

Statistical analysis was performed using the computer software SPSS and R (R Development Core Team, 2010). The significance level was set at 5%.

## Results

Tables 1a and b show incidence rates pmpy by age groups and time period. In all, 71 511 cases of cervical cancer were diagnosed and registered in women aged 20–79 years between 1982 and 2006. Only 36 cases were recorded in women aged 15–19 years, so no further analysis was performed in this age group.

Overall incidence of cervical cancer has nearly halved from 1982 to 2006 at an approximate annual rate of −3.2% in the time period 1982–1996 and at a greater rate of −3.9% in the time period 1992–2006 (*P* for trend <0.001) (Tables 1a and b). Analysis by 10-year age group showed that incidence rates have declined continuously in those aged 40–79 years (*P*<0.001). However, in contrast to all other groups, incidence of cervical cancer in women aged 20–29 years declined between 1982 and 1991, but increased significantly by 2.2% annually between 1992 and 2006. In women aged 30–39 years incidence decreased between 1987 and 2001 but there was no further decline after that (Table 1a).

**Figure 1:**
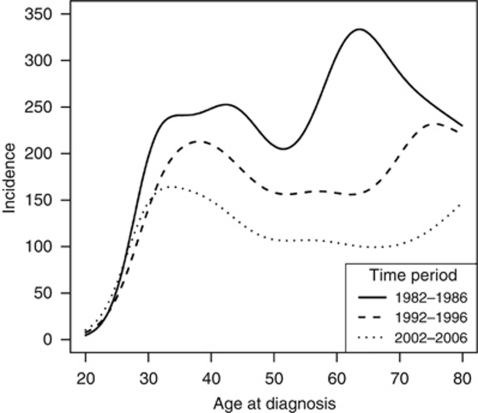
Smoothed incidence rate (per million person years) of cervical cancer in England by time period and age.

Figure 1 displays these results graphically for three 5-year time periods. It shows that there is a bimodal distribution in incidence with age in each period. However, the shape of the curve has changed over time. The age at maximum incidence of the first peak has declined from 40 years in the 1982–1986 period to 38 years for 1992–1996 and then to 33 years for 2002–2006. Conversely, the age at maximum incidence of the second peak has been displaced towards older ages by more than 10 years. In contrast to the earlier periods, during 2002–2006 the incidence at the younger age peak exceeds that at older ages.

**Figure 2:**
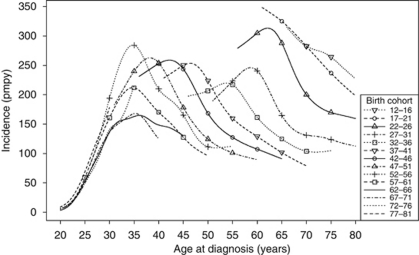
Smoothed incidence rate (per million person years) of cervical cancer in England by birth cohort and age.

**Figure 3:**
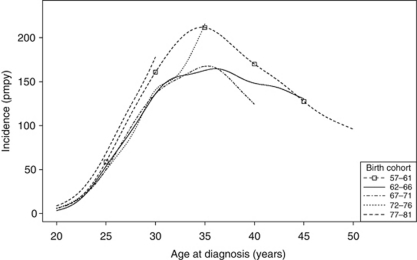
Smoothed incidence rate (per million person years) of cervical cancer in England by selected birth cohort (1957–1981) and age (20–49).

When the effect of birth cohort on incidence is considered (Table 2), it is clear that there are highly significant effects of both time period and birth cohort on the incidence rate by age group. Although the age–period–cohort model was significantly better than any of the simpler models (*P*<0.001), this model still had significant overdispersion, implying there were complex interactions between these effects. This can be seen in the plot of incidence by age for each birth cohort (Figure 2). The earliest and most recent birth cohorts (1902–1911 and 1982–1986) were excluded from the plot for clarity. The early birth cohorts (1922–1926 to 1947–1951) all have the same temporal pattern with an initial peak corresponding to 1988–1990 followed by a rapid decline. Birth cohorts from 1952–1956 to 1967–1971 all have peaks in incidence at around 35 years of age although the shape of the curve and overall incidence levels vary. In the 1972–1976 birth cohort the incidence rises more steeply after age 32 compared with 1962–1966 and 1967–1971. The incidence below age 30 in the most recent birth cohort (1977–1981) is clearly higher than in the birth cohorts between 1962 and 1976 (Figure 3).

Table 3a shows that there is significant regional variation in the incidence of cervical cancer for all time periods (*P*<0.001). Overall, the highest rate is seen in Yorkshire and the Humber, followed by North West, North East and West Midlands. The lowest rate is found in the East of England, followed by the South East and London GORs. Over the five time periods the three lowest ranked regions remained the same (London, South East, East of England). However, trends in incidence varied significantly by GOR as did the APC during 1982–1996 and 1992–2006, *P* in all cases <0.001 (Table 3b). East Midlands increased in rank and the North West decreased in rank over the time period of the study reflecting the low APC (−1.31) observed in the East Midlands in 1992–2006 and the respective high APC (−5.34) in the North West.

As trends over time among women aged 20–39 years differed from those aged 40 years and above, further analyses were carried out in this younger age group. Table 4a shows incidence in 20–29-year-olds by time period and GOR. In each time period, the rates varied significantly between regions. In 2002–2006, the highest rates were identified in the North East and Yorkshire and the Humber but London had a strikingly low incidence rate. The trends also varied by GOR (*P*<0.001). Six out of nine regions showed an initial decrease followed by an increase. This change was statistically significant in 5 regions. The exceptions were the North East, which initially had stable rates followed by a rapid increase, and the East of England, West Midlands and North West, which had fairly stable rates throughout (Table 4b). In North East, Yorkshire and the Humber, East Midlands and South West incidence rates have increased significantly from 1992 to 2006 with annual increases of 6.0%, 5.1%, 8.5% and 3.2%, respectively.

Table 5a shows incidence in 30–39-year-olds by time period and GOR. In each time period, the rates varied significantly between regions and the trend over time varied significantly by region (Table 5b). The trend during 1982–1996 compared with 1992–2006 was similar in most regions. However, in the East Midlands there was a significant decline in the first period but a significant increase during the later period. In the North West and West Midlands, the initial small decline in rates accelerated over time. As seen in 20–29-year-olds, the lowest rates in 30–39-year-olds were seen in London throughout the time period of the study.

As expected, incidence increased with increasing IMD quintile in both 20–29 and 30–39 year age groups (*P* for trend <0.001 in both age groups). However, when the differences in incidence by both GOR and deprivation for 20–39-year-olds during the period 1997–2006 (which is the only period for which the IMD was available) were studied, it was found that the effect of GOR was still significant (*P*<0.001) after taking deprivation into account.

## Discussion

This is the first study to show that, in 20–29-year-olds from 1996 onwards, incidence of cervical cancer is rising in most regions in England and incidence rates in women aged 30–39 years have mainly stabilised. In contrast, incidence has declined markedly in those aged 40–79 years. These differing trends between age groups have resulted in a marked change in the shape of the age–incidence curve for cervical cancer over time, such that the dominant peak in incidence is now below 40 years of age. Overall, incidence of cervical cancer in women aged 20–79 years in England has almost halved from 1982 to 2006. The decline is seen across all regions of England although there is significant regional variation in incidence in each time period. The greatest decreases in incidence rates were seen between 1987 and 1996, following the introduction of the formalised screening programme in 1988.

The changes to the shape of the age–incidence curve over time are best explained by a birth cohort effect. The peak age of incidence occurs progressively earlier in successive 5-year birth cohorts between 1912–1916 and 1952–1956 and declines from about 63 years of age to 35 years. Women born before 1923 would have been too old to be included in the formalised call–recall screening system. For cohorts born in 1922–1951 peak ages of incidence correlate with presumed age of first screening invitation, ranging from 60–64 years and 35–39 years, occurring within a year or two of this. Those included in the 1952–1956 birth cohort would have received their first screening invitation mainly before age 35 years and age of peak incidence stabilises at 35 years of age in the cohorts born in 1952–1971. Although, due to insufficient years of follow-up, it is not possible to determine age or magnitude of peak incidence in the two youngest cohorts, it is clear that incidence below age 30 years has increased markedly in the 1977–1981 birth cohort compared with the previous three cohorts (1972–1976, 1967–1971, 1962–1966) with most of the increase between ages 25 and 29 years. This youngest cohort was below 30 years of age at the end of the present study period. Whether the increased incidence of cervical cancer among this cohort of women will continue as they reach their 30s is not known. However, an increase in incidence around age 32–35 years is apparent in the 1972–1976 birth cohort corresponding to the same calendar years as the increase observed in the younger cohort.

Women born in 1977–1981 would have received their first screening invitation at age 20 years and first recall before 2005 when age of first screening invite was raised to 25 years. Although cervical screening is very effective in older women, screening is problematic in women aged less than 25 years with more unnecessary interventions being performed on lesions that may resolve (Moscicki, 2005, Moscicki *et al*, 2004, 2008). Furthermore, screening in this age group has little or no impact on cervix cancer incidence rates (Sasieni *et al*, 2009). Women who are screened at age 25–29 years and found to have cervical intraepithelial neoplasia grade 2/3 (CIN2, CIN3) are generally successfully treated. However, cervical screening coverage is lower in women aged under 40 years than in those aged 50–64 years. This difference is particularly marked in the youngest age group. In 2008, coverage in 25–29-year-olds was 58.6% compared with 82.2% in 50–54-year-olds. In this age group, coverage was nearly 7% higher in 1999 than in 2008, but in 2009 there was an increase of nearly 3% over the 2008 rate (NHS Information Centre, 2009). It remains to be seen whether this recent increase in coverage among 25–29-year-olds is maintained but efforts to further increase screening coverage in young women could help to limit the rising trend in cervical cancer incidence in this age group.

Human papilloma virus infection is necessary but not sufficient for development of cervical cancer, and other factors affect risk of HPV infections progressing to CIN. Human papilloma virus is sexually transmitted and aspects of sexual behaviour and reproductive factors affect risk of CIN and invasive cancer. These include number of sexual partners, age at first intercourse, early age at first full-term pregnancy and increasing duration of oral contraceptive use (International Collaboration of Epidemiological Studies of Cervical Cancer, 2006a, 2007, 2009). In 1998, 26% of UK female teenagers first had sexual intercourse under 16 years compared with just 4% in 1964 and sexually transmitted infections rates in 16–24-year-old women have more than doubled from 1995 to 2001 in the UK (Tripp and Viner, 2005). A recent government report shows that in 2009, in the UK, the peak age for sexually transmitted infections (STIs) in women is between 19 and 20 years, and that one in ten 15–24-year-olds diagnosed with an STI will become re-infected within a year (Health Protection Agency, 2010). The developing cervix at puberty and the healing cervix after delivery or any concomitant STIs pose a higher risk that HPV will reach the basal layer of the cervical epithelium, predisposing to a prolonged infection (Bosch *et al*, 2002).

A significant risk factor for squamous cell carcinoma of the cervix is tobacco smoking. Risk increases with number of cigarettes per day and younger age at starting smoking (International Collaboration of Epidemiological Studies of Cervical Cancer, 2006b). In Great Britain, the highest rates of smoking during 2005–2009 were seen in 20–24-year-olds. In this age group 30% are smokers and this rate has changed little in recent years although there has been a decline at older ages. About 20% of 16–19-year-olds smoke. The highest rates of smoking among women are found in the Yorkshire and the Humber, North East, North West and South West GORs (Office for National Statistics, 2010b), regions that also have high rates of cervix cancer. Changes in risk behaviours including smoking and sexual behaviour in women born from the 1970s onwards may have contributed to the rising incidence of cervical cancer in young women.

Previous studies have shown a link between social deprivation and increased incidence of cervical cancer (Hemminki *et al*, 2001; Singh *et al*, 2004; Shack *et al*, 2008). Shack *et al* (2008) examined socioeconomic variations in the incidence of cervical cancer between 1998 and 2003 by region and age groups over and under 65 years and concluded that 28% of cervical cancer cases annually could be prevented if incidence across the country could be reduced to that seen in the least deprived areas. In the present study, incidence among 20–39-year-olds in the least deprived quintile was 70% of that in the most deprived. However, our results demonstrated that deprivation alone could not explain the variability in incidence between geographical regions. Social deprivation may predispose to cervical cancer indirectly via increased prevalence of smoking, oral contraceptive use and decreased screening uptake. Geographical variations in incidence in younger women may be determined, in part, by geographical variations in smoking and sexual behaviour across deprivation categories as well as ethnic mix and levels of education (Health Protection Agency, 2010; Office for National Statistics, 2011).

A recent study has predicted a 76% reduction in lifetime risk of cervical cancer in 12-year-olds vaccinated against HPV 16 and 18 in the UK (Kohli *et al*, 2007). Human papilloma virus vaccination against the oncogenic high-risk strains 16 and 18 was introduced in England from 2008 for all girls aged 12–13 years. In addition, at the outset, a catch-up vaccination programme was introduced for girls up to the age of 18 years (National Health Service, 2008). It will be at least 10 years before the protective effect of vaccination has an impact on cervical cancer rates. In the meantime, screening offers a means of detecting and curing pre-invasive lesions and of limiting the rising incidence of cervical cancer in young women.

In conclusion, in spite of falling overall incidence rates for cervical cancer in England, in recent years incidence in young women under 40 years of age is stable or increasing. Those born from 1972 onwards appear to be at greater risk. The pattern is most marked in the North East, Yorkshire and the Humber and East Midlands GORs, which have higher rates of cervical cancer generally. These results have implications for implementation of public health programmes including measures to increase coverage of cervical screening, HPV vaccination and other public health interventions targeting young people such as sexual health education and tobacco control.

## Figures and Tables

**Table 1a:** Age-standardised incidence rate per million person years at risk and total number of cases (*N*) of cervical cancer in England by time period and age group

**Age (years)**	**1982–1986**	**1987–1991**	**1992–1996**	**1997–2001**	**2002–2006**	**1982–2006**	***N***
20–29	63	54	51	54	62	56	4943
30–39	234	245	191	158	159	195	17 289
40–49	238	249	185	161	127	188	14 965
50–59	231	223	157	123	106	164	11 430
60–69	318	277	164	129	102	201	12 320
70–79	263	266	222	166	116	208	10 564
20–79	213	208	154	128	112	161	71 511

**Table 1b:** Estimate of the annual percent change (95% confidence interval) and *P*-values for constant or different trends in incidence during 1982–2006

	***P*-values**		**APC**	
**Age (years)**	**Trend**	**Trend varies**	**1982–1996**	**1992–2006**
20–29	0.95	<0.001	−2.11 (−2.93, −1.28)	2.16 (1.29, 3.04)
30–39	<0.001	0.66	−2.32 (−2.74, −1.89)	−2.49 (−2.95, −2.03)
40–49	<0.001	<0.001	−2.41 (−2.87, −1.96)	−4.22 (−4.71, −3.73)
50–59	<0.001	0.15	−3.88 (−4.38, −3.38)	−4.58 (−5.16, −4.00)
60–69	<0.001	0.6	−6.01 (−6.47, −5.55)	−5.76 (−6.37, −5.14)
70–79	<0.001	<0.001	−1.50 (−2.02, −0.97)	−6.36 (−6.97, −5.74)
20–79	<0.001	<0.001	−3.19 (−3.39, −2.99)	−3.86 (−4.09, −3.62)

Abbreviation: APC**=**annual percentage change.

**Table 2:** Results of fitting age–period–cohort Poisson regression model to incidence of cervical cancer

**Model**	**Degrees of freedom**	**Deviance**	***P*-value compared to simpler model**	**Simpler model**
Null	119	18 690		
Age	108	6348	<0.0001	Null
Age+drift	107	4844	<0.0001	Age
Age+period	104	1664	<0.0001	Age+drift
Age+cohort	92	1029	<0.0001	Age+drift
Age+cohort+period	88	601	<0.0001	Age+cohort
Age+cohort+period	88	601	<0.0001	Age+period

**Table 3a:** Age-standardised incidence rate per million person years at risk and actual number of cases (*N*) of cervical cancer in England by time period and region for 20–79-year-olds

**GOR**	**1982–1986**	**1987–1991**	**1992–1996**	**1997–2001**	**2002–2006**	**1982–2006**	***N***
North East	224	227	170	155	133	181	4338
North West	251	255	200	152	125	195	12 254
Yorkshire and Humber	275	268	190	163	145	206	9279
East Midlands	218	194	137	127	132	159	5901
West Midlands	233	229	177	146	123	180	8451
East of England	164	166	124	91	80	123	5933
London	177	170	138	113	99	138	8534
South East	182	179	127	100	95	135	9630
South West	211	210	134	142	123	162	7191
*P*-value heterogeneity	<0.001	<0.001	<0.001	<0.001	<0.001	<0.001	

Abbreviation: GOR=Government office Region.

**Table 3b:** Estimate of the annual percent change (95% confidence interval) and *P*-values for constant or different trends in incidence during 1982–2006

	***P*-values**		**APC**	
**GOR**	**Trend**	**Trend varies**	**1982–1996**	**1992–2006**
North East	<0.001	0.27	−2.53 (−3.36, −1.70)	−3.42 (−4.35, −2.47)
North West	<0.001	<0.001	−2.31 (−2.80, −1.82)	−5.34 (−5.91, −4.77)
Yorkshire and Humber	<0.001	0.91	−3.54 (−4.10, −2.98)	−3.60 (−4.25, −2.95)
East Midlands	<0.001	<0.001	−4.62 (−5.32, −3.90)	−1.31 (−2.11, −0.49)
West Midlands	<0.001	0.007	−2.69 (−3.28, −2.10)	−4.21 (−4.89, −3.53)
East of England	<0.001	<0.001	−2.99 (−3.69, −2.29)	−5.31 (−6.11, −4.50)
London	<0.001	0.11	−2.59 (−3.18, −1.99)	−3.50 (−4.17, −2.83)
South East	<0.001	0.6	−3.63 (−4.18, −3.08)	−3.91 (−4.55, −3.28)
South West	<0.001	0.01	−3.89 (−4.53, −3.24)	−2.23 (−2.96, −1.50)
Trend varies by GOR	<0.001		<0.001	<0.001

Abbreviations: APC**=**annual percentage change; GOR=Government office Region.

**Table 4a:** Age-standardised incidence rate per million person years at risk and actual number of cases (*N*) of cervical cancer in England by time period and region for 20–29-year-olds

**GOR**	**1982–1986**	**1987–1991**	**1992–1996**	**1997–2001**	**2002–2006**	**1982–2006**	***N***
North East	59	57	57	96	103	72	324
North West	59	57	77	52	72	63	752
Yorkshire and Humber	98	64	57	75	88	75	656
East Midlands	78	46	38	65	81	60	423
West Midlands	75	83	76	82	71	78	709
East of England	47	36	50	37	53	44	400
London	40	35	23	32	29	31	524
South East	62	45	46	41	54	49	655
South West	62	79	51	57	81	65	500
*P*-value heterogeneity	<0.001	<0.001	<0.001	<0.001	<0.001	<0.001	

Abbreviation: GOR=Government office Region.

**Table 4b:** Estimate of the annual percent change (95% confidence interval) and *P*-values for constant or different trends in incidence during 1982–2006

	***P*-values**		**APC**	
**GOR**	**Trend**	**Trend varies**	**1982–1996**	**1992–2006**
North East	<0.001	0.09	0.80 (−2.67, 4.40)	6.04 (2.73, 9.46)
North West	0.21	0.2	1.91 (−0.26, 4.13)	−0.60 (−2.74, 1.60)
Yorkshire and Humber	0.64	<0.001	−5.10 (−7.28, −2.88)	5.05 (2.57, 7.59)
East Midlands	0.25	<0.001	−6.40 (−9.15, −3.57)	8.48 (5.32, 11.73)
West Midlands	0.76	0.5	0.49 (−1.69, 2.71)	−0.85 (−3.09, 1.44)
East of England	0.44	0.53	−0.29 (−3.23, 2.74)	1.41 (−1.55, 4.45)
London	0.01	0.01	−4.45 (−6.89, −1.96)	1.44 (−1.20, 4.16)
South East	0.12	0.01	−3.52 (−5.70, −1.29)	2.04 (−0.38, 4.52)
South West	0.53	0.02	−2.32 (−4.89, 0.32)	3.23 (0.50, 6.03)
Trend varies by GOR	<0.001		<0.001	<0.001

Abbreviations: APC**=**annual percentage change; GOR=Government office Region.

**Table 5a:** Age-standardised incidence rate per million person years at risk and actual number of cases (*N*) of cervical cancer in England by time period and region for 30–39-year-olds

**GOR**	**1982–1986**	**1987–1991**	**1992–1996**	**1997–2001**	**2002–2006**	**1982–2006**	***N***
North East	262	247	237	202	201	229	1055
North West	247	297	243	164	161	220	2675
Yorkshire and Humber	334	347	240	228	220	271	2392
East Midlands	238	213	175	175	220	203	1505
West Midlands	283	331	255	189	179	244	2272
East of England	178	191	165	116	124	153	1466
London	169	162	131	98	95	127	1828
South East	204	205	153	130	145	165	2349
South West	249	254	178	208	180	212	1747
*P*-value heterogeneity	<0.001	<0.001	<0.001	<0.001	<0.001	<0.001	

Abbreviation: GOR=Government office Region.

**Table 5b:** Estimate of the annual percent change (95% confidence interval) and *P*-values for constant or different trends in incidence during 1982–2006

	***P*-values**		**APC**	
**GOR**	**Trend**	**Trend varies**	**1982–1996**	**1992–2006**
North East	0.001	0.81	−1.26 (−2.99, 0.50)	−1.65 (−3.50, 0.23)
North West	<0.001	<0.001	−0.73 (−1.79, 0.35)	−5.09 (−6.26, −3.92)
Yorkshire and Humber	<0.001	0.17	−3.20 (−4.32, −2.07)	−1.71 (−2.96, −0.45)
East Midlands	0.06	<0.001	−3.47 (−4.94, −1.97)	2.18 (0.61, 3.77)
West Midlands	<0.001	0.005	−1.39 (−2.54, −0.23)	−4.49 (−5.75, −3.20)
East of England	<0.001	0.12	−1.38 (−2.83, 0.09)	−3.51 (−5.06, −1.92)
London	<0.001	0.83	−3.07 (−4.36, −1.77)	−3.34 (−4.73, −1.93)
South East	<0.001	0.07	−3.23 (−4.37, −2.08)	−1.29 (−2.54, −0.02)
South West	<0.001	0.09	−2.75 (−4.09, −1.38)	−0.59 (−2.03, 0.87)
Trend varies by GOR	<0.001		0.001	<0.001

Abbreviations: APC**=**annual percentage change; GOR=Government office Region.
